# Dielectric magnetochiral anisotropy

**DOI:** 10.1038/s41467-022-31225-3

**Published:** 2022-06-22

**Authors:** Geert L. J. A. Rikken, Narcis Avarvari

**Affiliations:** 1 Laboratoire National des Champs Magnétiques Intenses UPR3228 CNRS/EMFL/INSA/UGA/UPS, Toulouse & Grenoble, France; 2 Univ Angers, CNRS, MOLTECH-Anjou, SFR MATRIX, F-49000 Angers, France

**Keywords:** Electronic properties and materials, Ferroelectrics and multiferroics

## Abstract

The combination of chirality and magnetism has steadily grown over the last decennia into an area of intense research. Magnetochiral anisotropy, chirality-induced spin-selectivity and helimagnetism are the most prominent phenomena resulting from this combination, touching different systems like topological (semi-)metals and insulators, quantum magnets, type II multiferroics and enantio-selective synthesis. As an extension to this area, we argue, based on symmetry arguments, that magnetochiral anisotropy will manifest itself in the displacement current in chiral dielectrics in a magnetic field. We confirm this conjecture by the experimental observation of very strong dielectric magnetochiral anisotropy near the ferroelectric phase transitions of triglycine sulfate and Rochelle salt, two of the oldest and most investigated chiral ferroelectrics. This effect opens a new playground in the study and characterisation of all chiral dielectrics. With our discovery, magnetochiral anisotropy now covers the (di)electrical properties of all condensed matter, from insulators to superconductors.

## Abstract (web summary)

The interplay between chirality and magnetism has been attracting much. Here, the authors show that the movement of bound charges in a chiral dielectric depends on the relative orientation between this movement and an external magnetic field.

## Introduction

Chirality is vital in many areas of physics, chemistry and biology, where entities exist in two non-superimposable forms (enantiomers), one being the mirror image of the other. Since the time of Pasteur, the interplay between chirality and magnetism has been attracting much attention as a source of emergent phenomena. Fundamental symmetry arguments show that when a chiral system is placed in a magnetic field, a whole new family of effects, called magnetochiral anisotropies (MChA), becomes allowed (for a recent review, see ref. ^1^). The first member of this family to be observed, optical MChA, corresponds to a difference in the absorption and refraction of unpolarized light propagating parallel or anti-parallel to the field^2,3^. Initially observed in the visible wavelength range^4–6^, its existence was later confirmed across the entire electromagnetic spectrum, from microwaves^7,8^ to X-rays^9,10^. The second member, electrical MChA, was experimentally observed in the electrical properties of bismuth helices^11^, carbon nanotubes^12^, bulk organic conductors^13^, metals^14,15^, semiconductors^16^, and superconductors^17^ as a resistance *R* that depends on the handedness of the conductor and on the relative orientation of electrical current **I** and magnetic field **B**, given to first order by *R*^D/L^(**B**, **I**) = *R*_0_(1 + *γ*^D/L^**B** ⋅ **I**) where *γ*^D^ = −*γ*^L^ refers to the right/left-handed enantiomer of the conductor. The latest addition to the MChA family, phonon MChA, was recently observed in the propagation of ultrasound^18^, further illustrating the universality of the phenomenon. MChA has become a prominent representative of the wider class of non-reciprocal transport phenomena in broken-symmetry systems, that play an import role in topological quantum systems and in Berry phase physics^19^. Here we will present a novel manifestation of MChA, namely in the displacement current in chiral dielectrics in a magnetic field. Chiral dielectrics represent an enormous materials family of large scientific and industrial interest; many catalysts, most drugs, and almost all bio-molecules (DNA, proteins, sugars, etc.) are in this family. Dielectric MChA opens a whole new window on these materials.

Dielectrics when submitted to a time-varying electric field **E**(*t*) will carry a displacement current density **J**(*t*) because of the movement of bound charges. This displacement current density is given to first order by ${{{{{{{{\bf{J}}}}}}}}}_{0}=\dot{{{{{{{{\bf{P}}}}}}}}}={\varepsilon }_{0}{{{{{{{{\boldsymbol{\chi }}}}}}}}}^{({{{{{{{\bf{1}}}}}}}})}\dot{{{{{{{{\bf{E}}}}}}}}}$ where **P** is the polarization density and ***χ***^(**1**)^ the permittivity tensor. We can write a second-order series expansion of the perturbed symmetry-allowed total displacement current density, incorporating a heuristic magnetochiral term and a conventional second harmonic generation (SHG) term, as

1 $${{{{{{{\bf{J}}}}}}}}={{{{{{{{\bf{J}}}}}}}}}_{0}(1+{\gamma }^{{\rm {D/L}}}{{{{{{{\bf{B}}}}}}}}\cdot {{{{{{{{\bf{J}}}}}}}}}_{0})+\frac{\partial }{\partial t}({{{{{{{{\boldsymbol{\varepsilon }}}}}}}}}_{0}{{{{{{{{\boldsymbol{\chi }}}}}}}}}^{({{{{{{{\bf{2}}}}}}}})}{{{{{{{\bf{EE}}}}}}}})$$

where ***χ***^(**2**)^ is the first-order hyperpolarizability tensor, allowed in all non-centrosymmetric media and independent of the magnetic field. This can be rewritten as

2 $${{{{{{{\bf{J}}}}}}}}={\varepsilon }_{0}{{{{{{{{\boldsymbol{\chi }}}}}}}}}^{({{{{{{{\bf{1}}}}}}}})}\dot{{{{{{{{\bf{E}}}}}}}}}(1+{\varepsilon }_{0}{\gamma }^{{\rm {D/L}}}{{{{{{{\bf{B}}}}}}}}\cdot {\chi }^{({{{{{{{\bf{1}}}}}}}})}\dot{{{{{{{{\bf{E}}}}}}}}})+2{{{{{{{{\boldsymbol{\varepsilon }}}}}}}}}_{0}{{{{{{{{\boldsymbol{\chi }}}}}}}}}^{({{{{{{{\bf{2}}}}}}}})}{{{{{{{\bf{E}}}}}}}}\dot{{{{{{{{\bf{E}}}}}}}}}$$

For an applied electric field oscillating at frequency *ω*, this current density will have components oscillating at *ω* and at 2*ω*. The 2*ω* component will have contributions from conventional SHG, proportional to *ω* and independent of **B**, and from displacement MChA, proportional to *ω*^2^ and linear in **B**. Equation (2) can be reformulated for the magnetic field-dependent polarization at 2*ω* in a notation common in non-linear optics: ${P}_{i}^{2\omega }(\omega ,B)={{{\Gamma }}}_{ijkl}^{{\rm {D/L}}}(\omega ){E}_{j}^{\omega }{E}_{k}^{\omega }{B}_{l}$ where Γ^D/L^(*ω*) is a time-odd pseudo-tensor of rank 4 which quantifies the MChA. In particular ${{{\Gamma }}}_{ijkl}^{{\rm {D}}}=-{{{\Gamma }}}_{ijkl}^{{\rm {L}}}$. This illustrates that displacement MChA is a form of magnetic field-induced second harmonic generation (MFISHG)^20^ and **Γ**^D/L^ is an enantio-selective second-order magneto-electric hyperpolarizability. MFISHG, although less established than electric field-induced SHG, has been studied in the optical domain^21^. A small difference between the MFISHG response of the two enantiomers of a chiral material was observed^22^ and the MFISHG enantio-selectivity for small chiral molecules has been calculated^23^. For the optical case, the non-linear response is purely electronic, whereas here the dielectric response comes from the movement of (parts of) molecules and to our knowledge, no MFISHG has ever been reported for this case, neither theoretically nor experimentally.

Like any hyperpolarizability, Γ^D/L^ will rapidly increase with increasing polarizability of the medium, which for displacement MChA translates to an increasing dielectric constant *ε*. We have therefore experimentally searched for displacement MChA in chiral dielectrics with a very large *ε*. Such values can be found near a structural phase transition involving a ferroelectric phase, with the electric field applied parallel to the polar axis. As it happens, some of the best known and widely used ferroelectric crystals have a chiral phase, like triglycine sulfate (TGS) and Rochelle salt (potassium–sodium–l-tartrate tetrahydrate, L-RS), as summarized in Table 1^24,25^. Whereas RS crystals contain chiral tartrate ions, whose handedness determines the handedness of the crystal in all its phases, TGS crystals are composed of achiral molecules and the handedness of the chiral room temperature phase is undefined, determined by random crystal defects, and the crystal breaks up in domains of opposite handedness. A unique handedness can be imposed throughout the entire crystal by cooling it from the achiral high temperature phase in a strong electric field along the (polar) $\hat{y}$-axis^26,27^.

**Table 1:** Low temperature space group (LT SG), Curie temperature (*T*_c_) and high temperature space group (HT SG) of chiral ferroelectric crystals.

Material	LT SG	*T*_c_ (K)	HT SG
TGS	P2_1_	321.7	P2/m
RS	P2_1_	297	P2_1_2_1_2

Formal symmetry analysis shows that all 4th rank pseudo-tensors are zero for the P2/m space group, but that many elements of such tensors are non-zero for the P2_1_ space group, and that in particular Γ_*yyyy*_ ≠ 0. A similar conclusion also holds for the P2_1_2_1_2 space group^28^. This validates our conjecture that MChA can exist in the displacement current of chiral dielectrics. Note in particular that the same analysis shows that Γ_*yyyy*_ = 0 for the *m* point group, which contains the non-centrosymmetric, non-chiral space groups related to the polar chiral P2_1_ space group, illustrating that it is the chirality, and not the polarity of the P2_1_ space group that is reponsible for the MFISHG in the displacement current, and thereby of displacement MChA in TGS.

**Fig. 1:**
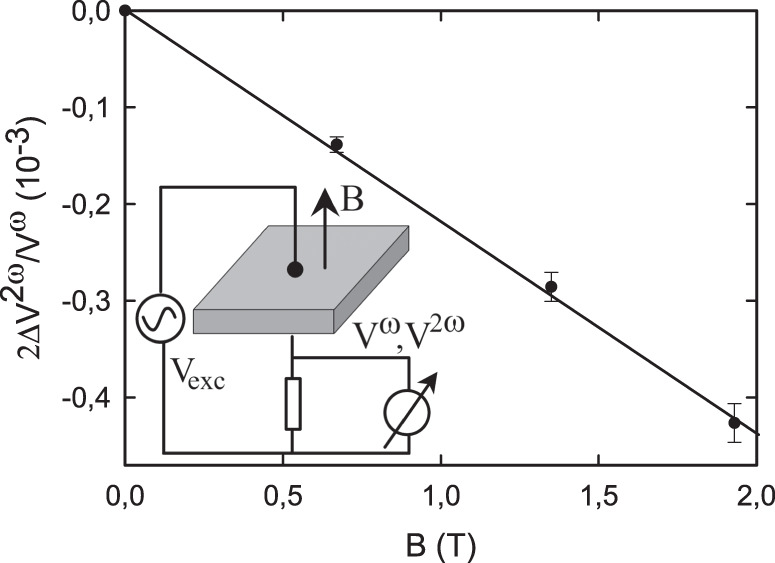
Field dependence. Magnetic field dependence of the MChA of a +poled TGS sample at 321 K and at 50 kHz and 10 V/mm. Solid line is a linear fit, error bars represent standard deviation on repeated measurement. Inset shows the schematic setup.

Our experimental observation of displacement MChA in chiral dielectrics consists of applying a periodic electric field smaller than the coercive field, and measuring the fundamental and second harmonic currents through the dielectric as voltages across a series resistor for both magnetic field directions (see inset Fig. 1 and see the “Methods” section). As samples, single crystals of poled TGS, L-RS and D-RS were studied close to their ferroelectric-paraelelectric phase transitions.

## Results

**Fig. 2:**
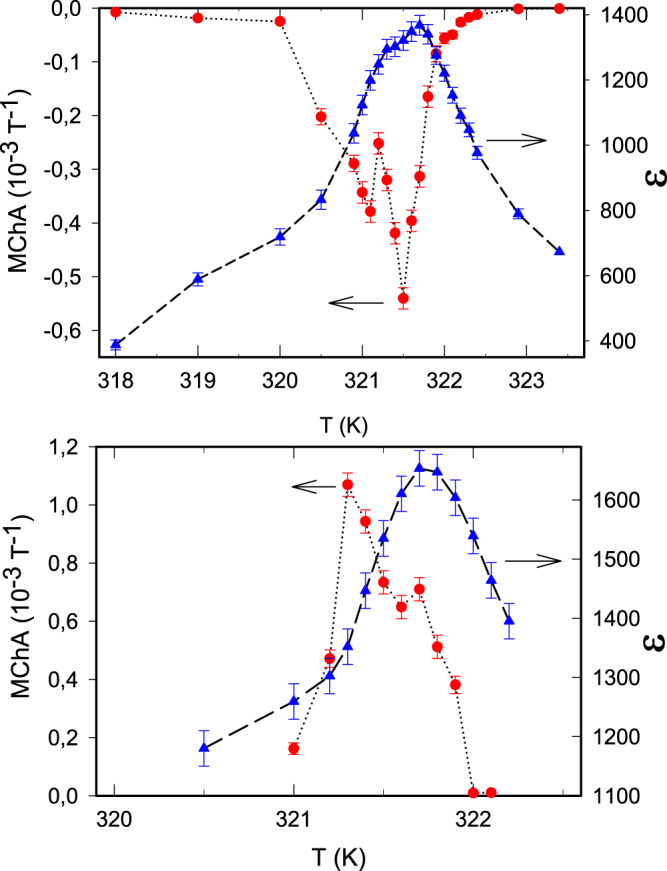
Chirality and temperature dependence. Temperature dependence of the dielectric constant (blue triangles) and the magnetochiral anistropy (MChA, red balls) of TGS samples. Top panel: +poled, bottom panel −poled. Electric field 10 kV/m, frequency 50 kHz, *B* = 2 T. Lines are guides to the eye.

**Fig. 3:**
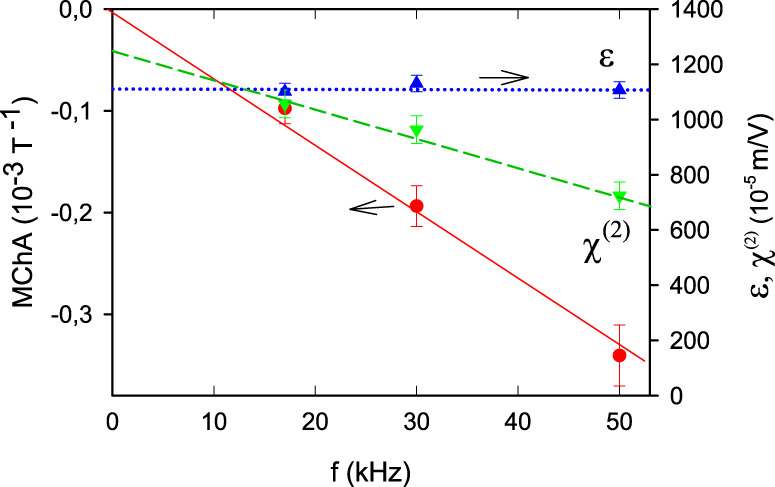
Frequency dependence. Frequency dependence of the magnetochiral anisotropy (MChA, red balls), the dielectric constant (blue up triangles) and the SHG hyperpolarizabiltiy (green down triangles) of a +poled TGS sample at 320.90 K. Electric field 10 V/mm, *B* = 2 T. Lines are linear fits.

Figure 1 shows the experimentally observed voltage ratio for a TGS crystal as a function of magnetic field at a temperature close to *T*_c_, confirming the existence of displacement MChA and its predicted linear magnetic field dependence. For electric fields along other crystal directions, the dielectric constant is much smaller and no MChA could be observed. For the remainder, we call 2Δ*V*^2*ω*^/*V*^*ω*^*B* the sample’s MChA. Figure 2 shows the observed MChA for two oppositely poled TGS samples, as a function of temperature close to *T*_c_, confirming that the poling procedure, which determines the handedness of the samples, determines the sign of the MChA. The maximum of the TGS MChA is well inside its chiral phase, at a lower temperature than the maximum in the dielectric constant, which identifies *T*_c_. The fine structure in the MChA peak suggests there is some inhomogeneity in the crystals. The MChA remains finite for at least 4 K below *T*_c_, and drops rapidly to zero in the paraelectric non-chiral phase above *T*_c_. This is fully consistent with the aforementioned symmetry analysis of Γ_*yyyy*_ for the P2_1_ and P2/m space groups and the second-order character of this phase transition which excludes discontinuities and hysteresis. The difference in the roles of chirality and of polarity is illustrated by the frequency dependence of the TGS (hyper)polarizabilities at a temperature close to *T*_c_, as shown in Fig. 3. This confirms that MChA is driven by the displacement current, which is proportional to the frequency, and not by the external electric field, and further validates our MChA conjecture. The observed linear frequency dependence of MChA will probably hold as long as the electric field period is below a characteristic dielectric response time of the medium, which for TGS is in the sub-microsecond range^29^.

**Fig. 4:**
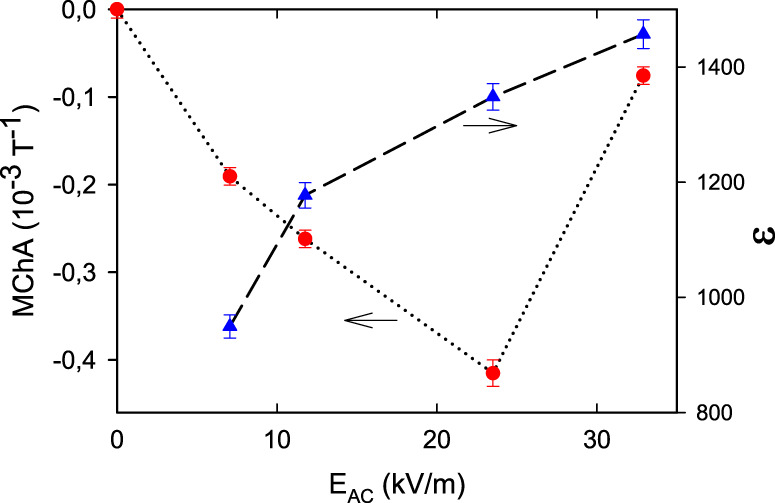
Electric field dependence. Excitation electric field dependence of the magnetochiral anisotropy (MChA, red balls) and dielectric constant (blue triangles) of a +poled TGS sample at 321.05 K at 50 kHz and *B* = 2 T. Lines are guides to the eye.

Figure 4 reports the electric field dependence of TGS MChA at a temperature close to *T*_c_ which shows a sub-linear dependence, followed by a breakdown. This is because at the highest applied electric fields, the field surpasses the coercive field, which is about 25 kV/m at room temperature, and which goes to zero at *T*_c_. Under these conditions, the sample changes handedness with the direction of the field, effectively annihilating the MChA signal.

**Fig. 5:**
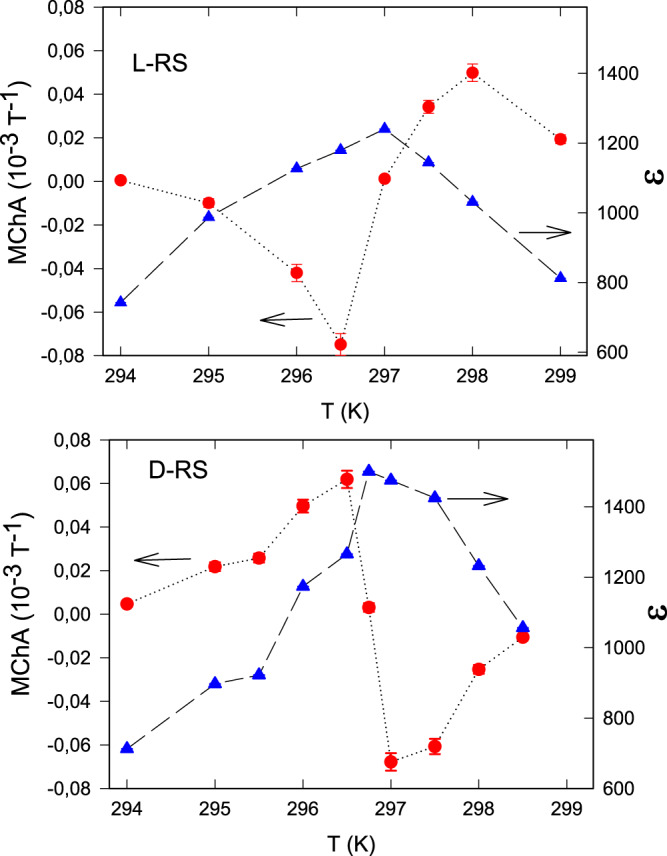
Chirality dependence RS. Magnetochiral anisotropy (MChA, red balls) and dielectric constant (blue triangles) of L-RS (top panel, filled symbols) and D-RS (bottom panel, open symbols) as a function of temperature. Lines are guides to the eye. Frequency 50 kHz, electric field 5 kV/m.

Figure 5 show the MChA for RS crystals, clearly showing opposite behavior for the two enantiomers. In contrast to TGS, where the MChA drops steeply to zero in the non-chiral phase above *T*_c_, the MChA in RS decreases more slowly above *T*_c_ because of the chirality of this phase. Again this proves that the MFISHG is due to the chirality and not due to the polarity of the crystal. The change in sign of the MChA at *T*_c_ is also observed for the all-electric second-order hyperpolarizibility of RS where it is explained by the classical phenomenological Landau–Ginzburg–Devonshire theory of ferroelectrics^30^.

## Discussion

Our experimental observations on dielectric MChA presented above confirm the existence of this effect and its basic characteristics. Expressing the displacement MChA in the form given by Eq. (2) allows for a comparison with the results for electrical MChA in metals or semiconductors that have been reported in the literature. Table 2 shows this comparison, showing that, quite surprisingly, dielectric MChA near the ferroelectric phase transition is orders of magnitude stronger than any resistive MChA ever reported, even those measured at low temperatures and in magnetic materials. This is most likely due to the collective ’soft’ character of the dielectric response near *T*_c_ of ferroelectrics in general and of TGS in particular, but so far no microscopic theory exists to confirm this. As the modern theory of polarization in general, and ferroelectrics in particular, is in terms of Berry phase physics, the latter may also apply here.

**Table 2:** Values for the magnetochiral anisotropy parameter reported here and in the literature.

Material	*γ*[*m*^2^/*T*⋅*A*]	Ref.	Remark
TGS	3 × 10^−5^	This work	Displacement
RS	3 × 10^−6^	This work	Displacement
t-Te	10^−8^	^16^	RT
Bi helix	3 × 10^−10^	^11^	77 K
TTF-ClO_4_	10^−10^	^13^	RT
CrNb_3_S_6_	10^−12^	^15^	Magnetic, LT
MnSi	2 × 10^−13^	^14^	Magnetic, LT
SWCNT	10^−14^	^12^	LT

A study of displacement MChA in chiral multi-ferroics may yield even higher values, as much higher magnetizations will exist in such materials as compared to the diamagnetic TGS and RS crystals. In particular in type II multiferroics, where chiral spin structures generate improper ferroelectricity, very rich and complex displacement MChA behavior can be expected. Other mechanisms that lead to large dielectric constants, like ionic conductivity or grain boundary effects in chiral media may also be able to generate measurable MChA. Observing dielectric MChA in ‘everyday’ chiral dielectrics, including liquids, with more common values of *ε*, typically in the range 5–30, is possible but will be challenging because of the expected small size of the effect due to its putative ${({{{{{{{{\boldsymbol{\chi }}}}}}}}}^{({{{{{{{\bf{1}}}}}}}})})}^{3}$ dependence, and will require the development of an appropriate sensitive measurement technique. However, if achieved, this would make displacement MChA a powerful and unique method to determine the handedness of chiral dielectrics with an electrical measurement, something of great analytical interest that so far requires much more complicated experimental techniques, ranging from polarization optics to labeled NMR. As displacement MChA is both sensitive to chirality and magnetization, and capacitive read-out is non-dissipative, other interesting applications in data storage, sensors and analysis can be envisaged. The existence of displacement MChA in dielectrics automatically means, as in the resistive case^31^, that inverse dielectric MChA exists; the generation of an enantio-selective magnetization by a displacement current, which may find applications in domains like spintronics or spin polarized ferroelectric electron emitters.

## Methods

**Fig. 6:**
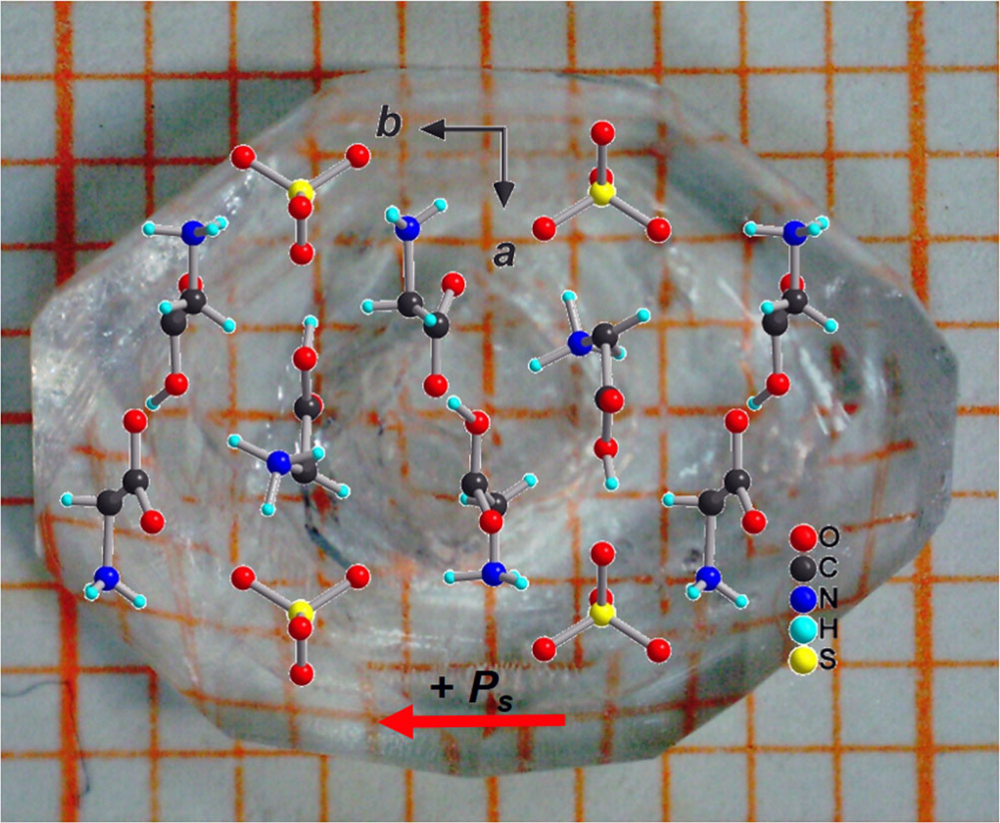
TGS crystal. View of an as grown TGS crystal with the molecular arrangement.

TGS and RS crystals were grown from aqueous solution by slow evaporation at room temperature over several weeks, cut into plates perpendicular to their polar axis ($\hat{y}$ for TGS (Fig. 6), $\hat{x}$ for RS) of typically 10 mm^2^ area and 0.6 mm thickness, and contacted with silverpaint. TGS plates were poled following the procedure of ref. ^26^. The handedness of the RS crystals follows from the handedness of the tartrate anions used in the synthesis. An AC voltage was applied across the sample and a series resistor, the latter translating the displacement current into a voltage. The fundamental and second harmonic displacement voltages were each measured by phase-sensitive detection with a lock-in amplifier for both magnetic field polarities and registered by a computer. The magnetic field was applied by an electromagnet, parallel to the electric field. The sample temperature was actively stabilized within 1 mK. The MChA of the samples is expressed as the difference between the second harmonic to fundamental voltage ratios for the two magnetic field directions:

3 $${{{\Gamma }}}_{iiii}{E}^{\omega }B=2\frac{{V}^{2\omega }(B)-{V}^{2\omega }(-B)}{{V}^{\omega }}\equiv 2{{\Delta }}{V}^{2\omega }/{V}^{\omega }$$

whereas the conventional SHG is given by the average of these two ratios.

## Data Availability

The data that support the findings of this study are available from the corresponding author upon reasonable request.
